# Energy, Carbon and Renewable Energy: Candidate Metrics for Green-Aware Routing?

**DOI:** 10.3390/s19132901

**Published:** 2019-06-30

**Authors:** Md. Mohaimenul Hossain, Jean-Philippe Georges, Eric Rondeau, Thierry Divoux

**Affiliations:** 1 Université de Lorraine, CRAN, UMR 7039, Campus Sciences, BP 70239, Vandoeuvre-lès-Nancy CEDEX 54506, France; 2 CNRS, CRAN, UMR 7039, France

**Keywords:** green networking, energy aware routing, carbon footprint, adaptive link rate, control and data plane

## Abstract

There are all sort of indications that Internet usage will go only upwards, resulting in an increase in energy consumption and CO_2_ emissions. At the same time, a significant amount of this carbon footprint corresponds to the information and communication technologies (ICT) sector, with around one third being due to networking. In this paper we have approached the problem of green networking from the point of view of sustainability. Here, alongside energy-aware routing, we have also introduced pollution-aware routing with environmental metrics like carbon emission factor and non-renewable energy usage percentage. We have proposed an algorithm based on these three candidate-metrics. Our algorithm provides optimum data and control planes for three different metrics which regulate the usage of different routers and adapt the bandwidth of the links while giving the traffic demand requirements utmost priority. We have made a comparison between these three metrics in order to show their impact on greening routing. The results show that for a particular scenario, our pollution-aware routing algorithm can reduce 36% and 20% of CO_2_ emissions compared to shortest path first and energy-based solutions, respectively.

## 1. Introduction

In this modern era of advancements, the ever-growing information and communication technologies (ICT) sector plays an important role. Improvement in technologies, the availability of inexpensive and extreme capacity optical transmission, increasing popularity of streaming services and Internet of things technology have increased the amount of usage of the Internet exponentially and there is no indication that this course will change. With the increasing dependency on ICT services, the need for generating electricity has also increased substantially in order to power the ICT infrastructure, which is a significant contributor in producing carbon dioxide, a leading greenhouse gas (GHG). Global GHG emissions data shows that the highest contributor to global CO_2_ emissions in 2015 was electricity production. Surprisingly, all the ICT devices and infrastructure, excluding smartphones, consumes 8% of the total electricity production and is projected to reach 14% by 2020 [1]. Similar claims are also stated into European Commission’s digital agenda report in 2013: “ICT products and services are currently responsible for 8 to 10% of the EU’s electricity consumption and up to 4% of its carbon emissions” [2].

Among the various ICT sectors, network architectures and devices are one of the highest energy consumers. According to [3], network devices and architecture are responsible of 29% of the total energy consumption by ICT. The main reason behind this is the increase of Internet users all over the world and secondly, most of the network architectures and resources like bandwidth, processing power and memory are designed bearing in mind the peak hour network usage [4,5]. This results in redundancy and overprovisioning of the resources and consumes extra energy during off peak hours which is unnecessary outside these peaks. 

Due to this ever-increasing energy consumption from ICT devices and especially from network infrastructures, it’s been a while now that researchers have started working on energy efficiency and hence the term “Green Networking” has emerged. The main idea behind green networking is to improve energy efficiency and reduce undesirable energy consumption which will in fact reduce the carbon footprint produced by ICT devices. Several works have been done considering the problem of energy efficiency in networks and there are mainly two research directions. The first at the network hardware level where the prime focus is on building energy efficient circuits, improving the power draining components like memory or modifying the cooling systems. The second direction is focused more on the network design level where the whole network architecture is considered, and optimization of the routing is done in order to reduce energy consumption also known as energy aware routing (EAR). EAR is implemented by different power optimization techniques like, turning off the unused network devices (nodes and links), Energy Efficient Ethernet (EEE) or Adaptive Link Rate (ALR). 

The main motivation behind working on energy efficiency is to reduce carbon emissions. However, it needs to be remembered that energy consumption is not always proportional to carbon emissions. Same amount of energy production can have a very different impact on the environment in terms of carbon emissions. For example, if we assume an average value of 400 g of CO_2_ emissions for 1 kWh of electricity production [6], France produces less than one fifth of the average value whereas Poland produces three times the average value. Therefore, for these two countries, consuming the same amount electricity has a quite different result in terms of environmental impact. Which begs the question: “*Is it enough to focus only on energy consumption while trying to achieve green networking?*”

**Figure 1:**
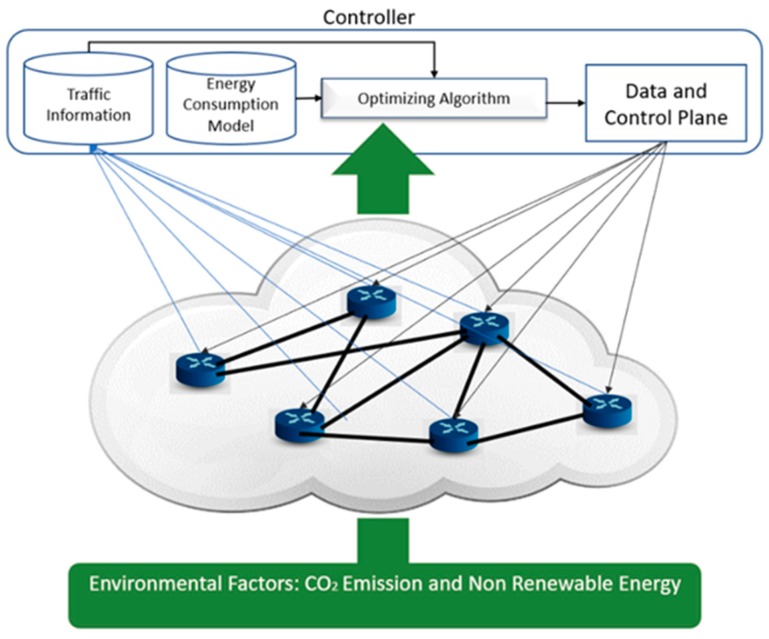
Basic Architecture of the System.

In order to address this question, in this paper, we have introduced the term pollution-aware routing (PAR) which uses energy efficiency techniques and environmental variables together to find a more pollution-aware solution. We have presented two different type of pollution metrics, namely carbon emission factor (CEF) and non-renewable energy usage percentage (NRE). Our idea is to compare and analyze the performance of the introduced pollution indicators with the well-known, widely used indicator, energy efficiency. For energy efficiency, we have considered two green policies. The first one is turning off the network devices (links and nodes) when they are not necessary and the second one is adaptive link rate (ALR). These policies are quite often used by the research community and very prominent in terms of energy savings. Section 2.1 contains a few of the works which have included these policies while designing energy aware routing. As explained in [7], the problem of finding the minimum number of links and nodes to be turned on while fulfilling the traffic constraint is a problem of multi-commodity flow class which is known as NP-hard problem. We have used a genetic algorithm as our heuristic to solve this problem. As a solution, we have provided a data plane and a control plane which contains the information of the routing and the bandwidth distribution of each link (ALR implementation), respectively. One main novelty of our algorithm consists of addressing both planes at the same time, leading to optimal solutions. In this paper, we have formulated the network and the energy consumption model for each of the network devices. By introducing CEF as a pollution indicator we want to address the carbon emission problem more directly. On the other hand, some energy resources have different impacts in addition to carbon emissions such as nuclear power plants, for example, produce nuclear waste. Therefore, our second pollution metric is NRE which will be applied to reduce the non-renewable energy usage percentage. The main difference between the currently used (i.e., energy consumption) and our introduced metric is that ours consider external factors (like power production) whereas energy consumption only reflects network efficiency. In Figure 1 we have depicted this phenomenon. Secondly, CEF and NRE will vary based on the location (i.e., country), in this regard we can say that we have addressed the problem with a more holistic approach considering in fact the political engagement of each country regarding the production of the energy.

Based on three different indicator/metrics three different objective functions are formulated, which are used as a fitness function for a genetic algorithm which is providing a set of data and control plane as output. These planes are providing operational topology for the network but by focusing either on reducing energy consumption, or total carbon emission or non-renewable energy usage percentage respectively.

We have considered our solution from the point of view of a software defined network (SDN). SDN is fairly a new network architecture where the data plane and control plane are separated in order to have more control over the network. It integrates a controller which orchestrates the underlying forwarding hardware. This centralized approach offers mechanisms allowing network operators to have a global point of view which is suitable for our scenario where a single change in one part of the network topology can have an impact all over the network. The authors of [8] used a centralized approach in order to provide zoning evaluation of voltage distribution. Moreover, a centralized system is always preferable for globally monitoring the system according to network policies and service requirements [9]. The controller receives the traffic information from the network, and based on the geographical position of the nodes, the values of environmental metrics like CO_2_ emission and non-renewable energy usage are received. Then the controller, based on the optimizing algorithm, provides a data plane and a control plane for the network. The detail description of how a control plane and a data plane are designed will be described in Section 3. Figure 1 gives an overview of the system. 

The paper is organized as follow: Section 2 explains the motivation in the light of related work and sustainable green networking and summarizes the objective of our work. Section 3 contains the concept of EAR and PAR and a description of centralized sustainable routing. Section 4 contains the formalization of the problem and the overview of the heuristic function. In S5 we have a case study and the performance evaluation of the algorithm and lastly Section 6 and Section 7 contain the future work and conclusions of the work, respectively.

## 2. Motivation

### 2.1. Related Work

For the last decade or so, several approaches are made in order to achieve green networking. The authors of [10] provided a detail discussion on the different research trends in green networking. Research works like [11,12] focus only turning off links and line cards whereas many other papers like [13,14,15] focus on turning off both links and nodes. Authors in reference [4] proposed a novel heuristic, GreenTE, which maximizes the power savings and satisfies the quality constraints including link utilization and packet delay. The proposed a heuristic solution that is proactive in terms of path calculation. They have defined a set of previously calculated paths and they chose the solution from one of them. Authors in references [12,13] emphasize least link and least flow without link adapting. They have used a greedy algorithm to find out the least used link or devices with the least flow and then turn them off. They did reactive path calculation. This means that based on the heuristic several paths are being calculated instantly and the best one for the particular scenario is chosen. Works like [15], provide a clear idea about how an optical core network behaves in terms of energy consumption. On the other hand [16,17,18] all talked about implementing ALR in order to reduce energy consumption. Other works like [12,13,19,20,21] all have considered energy aware routing. Authors in reference [20] provided a greedy algorithm-based heuristic and along with links they have also focused on reducing the energy consumption by turning off nodes. Nevertheless, in their solution they have not considered ALR. In current times alongside these solutions various other solutions have been proposed using SDN in order to benefit from the global knowledge of the central controller. Authors in reference [14] proposed an incremental greedy algorithm-based solution where a controller dynamically adds nodes and links to the initial small topology network in order to satisfy user demand requirements. Authors in reference [22] also proposed an energy efficient solution combining energy aware routing with SDN-based Ethernet networks. There are also a few works which have taken a different approach towards green networking. Authors in references [23,24,25] all have tried to achieve green networking in data centers by using renewable energy. Authors in references [23,24] changed the destination node based on availability of the renewable energy and on the other hand in [25], alongside with renewable energy, other criteria like geographical load balancing and server speed scaling are considered. The authors of [26,27] proposed a similar eco-friendly routing idea using renewable energy where they have used clustering to choose a cluster head with most renewable energy and then choose the member node accordingly. Authors in reference [26] is for wireless networks and [27] is for IP networks. However, one thing is common in the abovementioned works is that all of them consider green network that same as the energy efficient network. They have all tried actively or passively to reduce the energy consumption in order to get an energy efficient network. However, even if energy has a link to sustainability issues, its direct impact on the environment in terms of air pollution and Earth’s resources is not explicitly specified. Therefore, in our work we have introduced two different green metrics–CO_2_ emission and non-renewable energy usage percentage–and make a comparison between them and energy efficiency approaches.

### 2.2. Sustainability and Green Networking

The authors of [28] explained a very important point by mentioning that achieving sustainability is not a straightforward approach of saving resources like energy. Even if from technical point of view, it might seem direct approach, in fact the economical and behavioral perspective of the society needs to be included when trying to achieve a sustainable solution. In [29], they have explained that, in order to build a sustainable system, it is necessary to determine the shared interests of both business and society’s point of view. In fact, sustainability is a unified concept which considers environmental, social, and economic aspects as three fundamental pillars. They indicate that, viable development requires the understanding of nature, society and economic capital or colloquially speaking the planet, people, and profits [30,31,32]. Therefore, in order to design a sustainable green networking architecture, it is very important to somehow consider all the three pillars. In this paper, we have proposed a centralized model of generating sustainable control and data planes considering the three pillars. The details of the model are described in Section 3.2.

### 2.3. Summarizing the Goal

We have discussed a few works focusing on renewable energy, but almost all of them are focused on topology-aware routing, which is different from energy-aware routing. To the best of our knowledge, carbon emissions are not fully considered as a routing metric. In this regard, the goal of this paper is to compare the performance of these metrics and how algorithms (optimizing both routing and green policies) need to be adapted. Performance is going to be assessed in a centralized sustainable routing model.

## 3. Green Routing and Metrics

### 3.1. Concept of EAR and PAR

In order to get a clear view of the proposed system, it is better to start with EAR and PAR. While implementing green networking, one of the most common and effective solutions is to shut down as much network equipment as possible while keeping the operational network, which is also known as energy-aware routing (EAR). This drop in the number of active devices reduces the energy consumption which has in fact a positive impact on the carbon footprint. The fundamental difference between a classical routing approach like shortest path first (SPF) and EAR is that SPF considers the number of hops and tries to reach the destination as quickly as possible without focusing on the energy consumption of the network, whereas, EAR tries to reduce the overall energy consumption of the network, which might result a longer path for some demands and similar length paths for other demands. 

**Figure 2:**
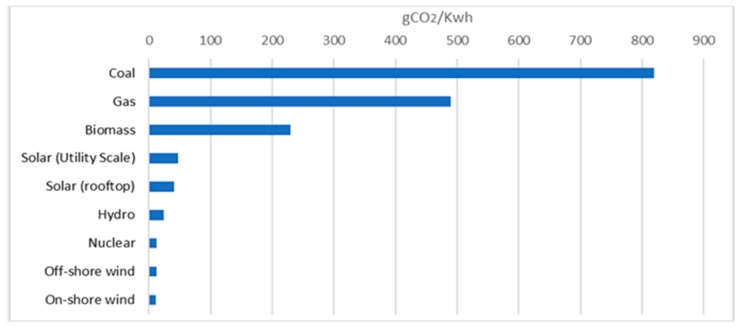
Emissions of selected electricity supply technologies (gCO_2_/kWh).

In this paper we have talked about a different type of routing strategy which is pollution-aware routing (PAR). The main concept of PAR is to route the demands with different paths based on environmental factors such as carbon emission factors and the non-renewable energy usage percentage of a node and try to reduce the targeted goal of reducing the CO_2_ emission or the usage percentage of non-renewable energy of the total network architecture. This routing is applicable only for geographically diverse, distributed network architectures where every node has different means of energy production. The main motivation behind PAR is the variation of values of CO_2_ emission in terms of energy production methods as shown in Figure 2. Values are taken from [33].

**Figure 3:**
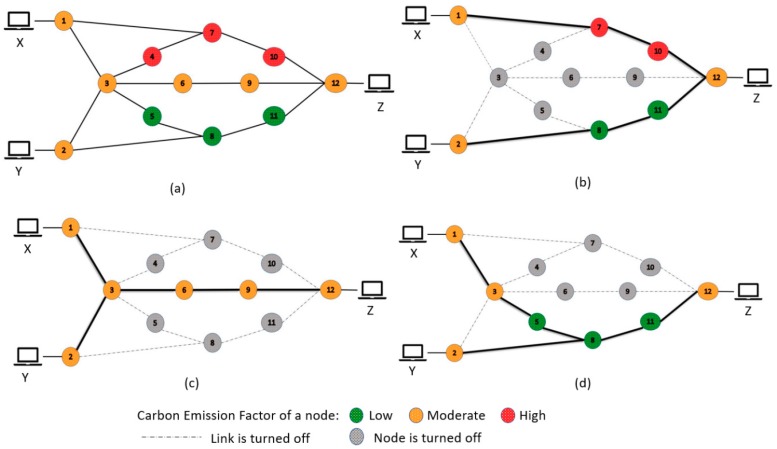
Example of SPF, EAR and PAR. (**a**) shows a topology with 12 nodes, (**b**) shows the topology for SPF, (**c**) shows how EAR will act in this scenario, (**d**) we have a different topology compared to (**b**,**c**).

It can be clearly seen that for same amount of electricity production the CO_2_ emissions can vary a lot. Therefore, in PAR, instead of reducing the energy consumption of the network architecture, the environmental metrics are considered. The first metric is CO_2_ emissions. As different countries use different techniques for producing electricity, they all have different carbon emission factors (CEFs). Therefore, in this case, the routing will be based on the CEF. The paths for the demands will be chosen in order to reduce the carbon emissions of the total network architecture. Similarly, here we have considered a second kind of PAR with the second green metric NRE, where nodes will be chosen based on renewable energy (wind, solar, hydro) percentage. The idea is to decrease the overall amount of non-renewable energy usage of the network architecture while fulfilling the demands. Both find the paths for the demands in order to facilitate their targeted goal without considering the path size or amount of energy consumption. The example depicted in Figure 3 will give a better grasp of the concept. 

Figure 3a shows a topology with 12 nodes. For this example, we have considered CO_2_ emissions as a factor for PAR. For simplicity there are only two demands. One is from X to Z and the second one is from Y to Z. Again, to avoid complexity, the sum of the two demand requirements is less than any available links’ bandwidth. Three different colors are used instead of values for the carbon emission factor. Green means the node has the lowest amount of CEF, orange means moderate and red means it has the highest amount of CEF. A node is grey colored if it is turned off. Figure 3b shows the topology for SPF. The lowest number of hops are considered for each demand. Regardless of the nodes CEF or even without considering the total energy consumption, Figure 3c shows how EAR will act in this scenario. It will choose a common link in order to reduce the number of active nodes and links. It will reduce one node and two links compared to SPF. However, this will also not be concerned with the CO_2_ emissions. In Figure 3d we have a different topology compared to Figure 3b,c. It didn’t look for the shortest path or lowest number of nodes and links. Rather it chooses a topology where the total amount of CO_2_ emissions will be low. It will not consider the total number of turned on nodes and links if the overall CO_2_ emissions are on the low side. 

### 3.2. Centralized Sustainable Routing

**Figure 4:**
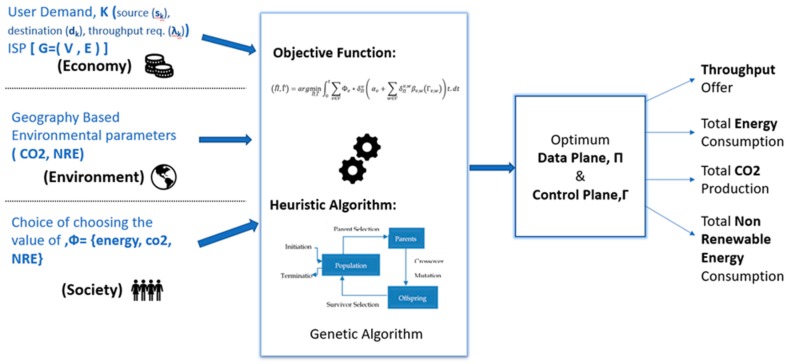
Generation of sustainable data and control plane.

In this paper, we have also proposed a sustainable solution for the problem of achieving ‘green networking’. As explained in Section 2, a solution cannot be sustainable without addressing the three pillars of sustainability. In the section above, EAR and PAR are described. Our solution, depicted in Figure 4, integrates all these elements in order to address the ‘green networking’ problem. Through our solution we would like to investigate the performance of the proposed approach (PAR) and the available approach (EAR). We have designed the solution keeping in mind the three pillars of sustainability, namely economy, environment and society. Figure 4 shows the architecture of the proposed system. In the following part, how the three pillars are integrated into our solution is described briefly. Firstly, our system takes traffic demand from users and the topology of the network from the Internet service provider (ISP) as input. Both users and ISPs are the stakeholders for our solution. ISP has a direct relation with the profit, whereas users are mainly concerned about the quality of service (QoS) like throughput requirements. We also take geography-based environmental information such as carbon emission factor (CEF) and non-renewable energy usage percentage (NRE) as input. This information is one of the key factors for providing a green outcome. These environmental parameters are acting as the second pillar of sustainability–environment. Lastly, our system provides a user of this system the ability to choose to select a solution focusing on either reducing energy consumption or CO_2_ emissions or non-renewable energy usage percentage (the value of Φ in Equation (8)). The social aspect of sustainability focuses on balancing the needs of the individual with the needs of the group. By giving the choosing ability to the Internet service providers, it integrates the social entity of the sustainability into our system. 

For example, in case of the GÉANT network, any environmental strategy or law defined and imposed by European Union can be implemented in the network by tuning the optimizing equation. After getting these inputs our system provides a solution based on the equation derived in the previous section. It uses a genetic algorithm as the heuristic algorithm in order to solve the optimization problem. The heuristic algorithm is described in the following section. After that an optimum data and control plane are provided to implement the solution into the topology. These data and control planes are calculated based on the inputs given to the system. The main goal of these planes is to minimize the given objective function while fulfilling the QoS constraint. Alongside the solution topology settings, these planes also provide vital information about energy consumption, CO_2_ emissions and non-renewable energy usage percentage of the topology. This information is essential, especially for the people pillar of sustainable development because it provides clear information about the impact of network users on the environment, which by default provides them a guideline to act accordingly. This kind of feedback always make the people in society more involved in the system which is a prerequisite for creating a complete sustainable system. Our proposed solution is a sustainable solution towards achieving green networking. The heuristic algorithm used for optimization is explained in the next section. 

## 4. Formalization of the Problem

### 4.1. Problem Definition

Let us assume a network topology is defined by a directed graph G = (V, E), where V is set of vertices i ∈ {1, 2, . . ., |V| = n} and E is set of edges (i, j), which are 2–elements subsets of V. The adjacency matrix is a square |*V*| × |*V*| matrix A, such that the element *A_i,_* ∈ {1,0} has the value of 1 when an edge exists between two vertices i and j, and 0 when there is no edge. The C matrix contains the capacity of each edge that means C_i j_ represents the capacity of the link between node *i* and *j*. Capacity is the only part of G which is controllable. Now let’s come to the dynamic part of the network which is traffic demands. Let’s suppose K is a set of traffic demands K = {1, 2, …, k}. A demand consists of three information source (s_k_), destination (d_k_) and throughput requirement (λ_k_) expressed in b/s. 

In the framework of Software-Defined Networking (SDN) and more generally speaking network automation, we would consider that the topology is redundant, which means that several paths (i.e., more nodes and links are added—offline—to the physical topology) are available. However, only those paths will be considered which are satisfying both throughput demand and the capacity constraint of the links. The data plane matrix is denoted by Π, which includes all the forwarding decision for all flows over all nodes. Each row of the matrix is dedicated for one demand. **Π_k,v_** returns the node **v + 1**, which is an adjacent node of **v** into path P_k_ (**Π_k, v_ = v + 1**) or null set if the path does not include node **v**. The path ends when it returns d_k_. A path $P_{k}=\left\{s_{k},\;\Pi_{k,s_{k}},\;\Pi_{k,\Pi_{k,s_{k}}},\;...,\;d_{k}\right\}$ then can be retrieved. As mentioned earlier, different paths are available for every pair of source and destination therefore for each demand set several data planes are possible. The data plane should respect the following constraint for a demand i:

(1) $$\forall v\in \{s_{i-}d_{i}\},\;\;w=\Pi_{i,v\;\;\;}such\;as\;\sum_{\left(k\epsilon {K|\Pi }_{k,v}=w\right)}\lambda_{k}\leq C_{v,w}$$

This ensures that only the links that are in the topology are considered and no link will be assigned more throughput than its capacity. Furthermore, the control plane, denoted by matrix Γ, is used to define the controllability of the link capacity (i.e., ALR mechanism). The capacity can be switched to different discrete values from the maximum capacity defined by C to 0. Zero is considered when both interfaces of a link are turned off. Initially, Γ = C and later from the second flow C is replaced by Γ in Equation (1). The value $\Gamma_{i,j}$ represents the final link capacity required to fulfill every demand requirement. 

**Figure 5:**
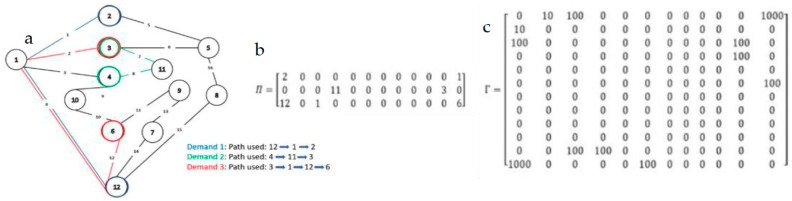
(**a**) Topology (a portion of the GÉANT network); (**b**) A possible data plane; (**c**) control plane.

Figure 5 shows an example of how data and control planes work. Here as a topology a portion of the GÉANT Network is used and let’s consider that the topology has three different levels of bandwidth: 10, 100 and 1000 Mbps, respectively. For three demands $K=\left\{\left\{s_{1}=12,\;d_{1}=2,\;\lambda_{1}=8\;\mathrm{Mb}/s\right\},\left\{s_{2}=4,\;d_{2}=3,\;\lambda_{2}=60\;\mathrm{Mb}/s\right\},\;\left\{s_{3}=3,\;d_{3}=6,\;\lambda_{3}=95\;\mathrm{Mb}/s\right\}\right\}.$ The three chosen paths are {12 – 1 – 2}, {4 – 11 – 3} and {3 – 1 – 12 – 6}, respectively. Then the data plane and the control plane will be look like Figure 5b,c. There are several possible solutions. The data plane is created based on the chosen paths and the control plane is created based on the data plane. Even though all the demands have a bandwidth requirement of less than 100 Mbps, we can see in the control plane, edge {1,12} (link 4 on Figure 5) has a final capacity of 1000 Mbps. In fact, this edge is included in the solution path of both demand-1 and demand-3 which have a combined throughput demand of 103 (8 + 95) Mbps. A link capacity of 100 Mbps is hence not anymore enough and as only three levels of bandwidth, namely 10, 100 and 1000 Mbps are considered, it will be replaced by a 1000 Mbps capacity one. Both data plane and control planes are vital for calculating our objective functions that will be discussed in the next part.

### 4.2. Modelling of the Objective Functions

We have three different criteria that need to be minimized, namely energy consumption, CO_2_ emissions and the non-renewable energy usage. We have formalized the cost of data and control planes based on the objective function. However, as mentioned earlier, the carbon emissions and non-renewable energy both can be used as a multiplicative factor with the energy model. Therefore, here the energy model is described first. For calculating the energy consumption, a simple model from [34] is used where energy consumption model of a Cisco Ethernet switch is provided. Similar models can also be found in the literature [8,35,36], even for different devices. To simplify, it is assumed here that energy consumption of a node **v**, follows the linear model:

(2) $$\varepsilon_{v}\left(t\right)=\overset{t}{\underset{0}{\int }}\left(\alpha_{v}+\underset{w\epsilon v}{\sum }\delta_{v,w}\beta_{v,w}\right)t\;.\;\;dt$$

where $\varepsilon_{v}$ in Watt-hour (Wh), α is the static power consumption (when no interfaces are activated), $\delta_{v,w}=1$ if $C_{v,w}>0$ and 0 else (to know if the interface to the neighbor w is used) and $\beta_{v,w}$ is the power consumption of the interface port itself. The model consists of two parts: a fixed/static one that must be considered each time the node is turning on and a dynamic one that depends on the control plane. As we want to include two green strategies (turn off nodes when not required and ALR) into the model without hampering QoS constraint, in order to imply the first strategy, two binary variables are included:

(3) $$\delta_{\Pi }^{v}=\{\begin{array}{c} 1\;if\;\sum_{k\in K}\Pi_{k,v}>0\;or\;v\in \Pi \\ 0\;\;\;\;\;\;\;\;\;\;\;\;\;\;\;\;\;\;\;\;\;\;\;\;\;\;\;\;\;\;\;\;\;\;\;\;\;\;\;\;\;\;\;\;\;else \end{array}$$

(4) $$\delta_{\Pi }^{v,w}=\{\begin{array}{c} 1\;if\;\exists_{k}\in K,\;\Pi_{k,v}=w\;or\;\Pi_{k,v}=v\\ 0\;\;\;\;\;\;\;\;\;\;\;\;\;\;\;\;\;\;\;\;\;\;\;\;\;\;\;\;\;\;\;\;\;\;\;\;\;\;\;\;\;\;\;\;\;\;\;\;\;\;\;\;\;\;else \end{array}$$

A node v is used in the data plane if it either forwards ($\sum_{k\in K}\Pi_{k,v}>0$) and receives (v∈Π) traffic or it is used as a mediatory node for a selected path. Now, in the case of ALR, the second policy that we have considered, the matrix Γ contains the decisions (Figure 5c), such the power consumption $\beta_{v,w}$ will now vary according to the link capacity. Hence, the objective function in terms of data and control planes while satisfying the demands requirements (elementary paths, throughput, links capacity limitations) is as follows:

(5) $$\left(\hat{\Pi },\hat{\Gamma }\right)=\arg\underset{\hat{\Pi },\hat{\Gamma }}{\min}\int_{0}^{t}\underset{v\in V}{\sum }\delta_{\Pi }^{v}\left(\alpha_{v}+\underset{w\in V}{\sum }\delta_{\Pi }^{v,w}\beta_{v,w}\left(\Gamma_{v,w}\right)\right)t.dt$$

This objective function depicts the problem of minimizing the energy consumption of overall system. Now, let’s consider the environmental factors. Suppose, Λ is a set of carbon emission factors (CEFs) for all the nodes. Where, $\Lambda =\left\{\Lambda_{1,}\Lambda_{2},\ldots ,\;\Lambda_{v}\right\}$. And ψ is a set of non-renewable energy usage percentage (NREs) for all the nodes. $\psi =\left\{\psi_{1,}\psi_{2},\ldots ,\;\psi_{v}\right\}$. Here one thing to notice is that for all, the goal is to reduce it. 

As mentioned earlier, that both environmental parameters are a multiplicative factor of the original energy consumption model, therefore, objective function can be rewritten for CEF and NRE respectively in the following way:

(6) $$\left(\hat{\Pi },\hat{\Gamma }\right)=\arg\underset{\hat{\Pi },\hat{\Gamma }}{\min}\int_{0}^{t}\underset{v\in V}{\sum }\Lambda_{v}*\delta_{\Pi }^{v}\left(\alpha_{v}+\underset{w\in V}{\sum }\delta_{\Pi }^{v,w}\beta_{v,w}\left(\Gamma_{v,w}\right)\right)t.dt$$

(7) $$\left(\hat{\Pi },\hat{\Gamma }\right)=\arg\underset{\hat{\Pi },\hat{\Gamma }}{\min}\int_{0}^{t}\underset{v\in V}{\sum }\psi_{v}*\delta_{\Pi }^{v}\left(\alpha_{v}+\underset{w\in V}{\sum }\delta_{\Pi }^{v,w}\beta_{v,w}\left(\Gamma_{v,w}\right)\right)t.dt$$

The goal is to provide data and control planes which will reduce the energy consumption or carbon emission or non-renewable energy percentage of the overall system. Then from Equations (5)–(7) a generalized version of the objective function for system would be:

(8) $$\left(\hat{\Pi },\hat{\Gamma }\right)=\arg\underset{\hat{\Pi },\hat{\Gamma }}{\min}\int_{0}^{t}\underset{v\in V}{\sum }\Phi_{v}*\delta_{\Pi }^{v}\left(\alpha_{v}+\underset{w\in V}{\sum }\delta_{\Pi }^{v,w}\beta_{v,w}\left(\Gamma_{v,w}\right)\right)t.dt$$

where, $\Phi_{v}=1\;or\;\Lambda_{v}\;or\;\psi_{v}$ depending on the minimizing criteria (i.e.,: energy consumption, carbon emission, non-renewable energy percentage) of the system.

### 4.3. Description of the Heuristic Function

This optimization problem falls into the multi-commodity flow class which is known as NP-hard problems and a heuristic algorithm is required to find the solution. Therefore, in order to solve our optimization problem formulated in the previous section we have used a genetic algorithm (GA) as our heuristic algorithm. It is an evolutionary algorithm which is inspired by the genetic processes of biological organisms. In [37,38,39] it is shown how this heuristic can be applied for solving NP-complete optimization problem related to networking like finding shortest path or designing an industrial Ethernet infrastructure. A GA has crossover and mutation which help the problem to not to get stuck in local minima which makes it robust than any other enumerative approaches. In the following part different parts of the GA are described in brief.

**Figure 6:**
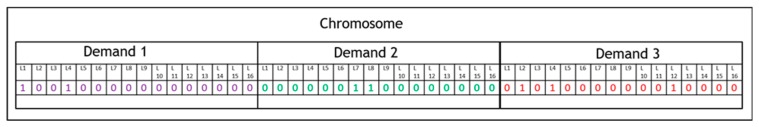
Structure of the chromosome.

The crucial part of applying GA to a problem is to design a chromosome according to the problem. Later these will create a population pool. Each chromosome represents a single candidate solution of the search space. In our case, the data plane is converted into a chromosome as a data plane contains a solution set for each demand following the capacity constraint. Therefore, in order to build a chromosome pool, we have created a set of data planes. To do that, we have used another heuristic which is randomized depth first search (RDFS). RDFS randomly selects the next node for going into next level into the tree. Simple DFS is unable to create a diverse solution set. Therefore, RDFS is used to find the path for each demand and then that path is added to create the solution set. RDFS considers the current state of the topology capacity wise. Then, all the solutions that are found, follow the capacity constraint. Once we have a data plane then we can convert the data plane into chromosome. If we have a set of demand k = {1, 2, 3, …, k} and if a topology has E number of edges {1, 2, 3, …, E} then in a chromosome consists of k*E number of 0’s and 1’s. Where 0 represents this link is not used for fulfilling this demand and 1 represents this link is turned on for fulfilling this demand. Figure 6 shows the structure of the chromosome. This chromosome is actual representation of the example with three demands that we discussed in Section 4.1. In our initial population pool, we have always included one solution achieved by using SPF. Therefore, a population pool with a size of N has N-1 chromosome gathered by RDFS and the other one is using SPF. 

For crossover operation we have decided to randomly select half of the bits of strings from one chromosome and rest half from another chromosome instead of using one cut or two cuts where chromosomes are divided into two equal parts or three equal parts and change the parts in between them respectively. By doing single crossover operations the process is generating two new chromosomes. In our case mutation is also done demand wise. Instead of a single bit values for a single demand, the whole strings of bit of a demand is replaced. There are two kinds of mutation percentage. The first one indicates how many of the chromosomes which are previously shortlisted by selection process will go through the mutation process. However, from a chromosome, how many demands will be selected for mutation is denoted by the second one. For example, mutation-1 equals to 10% means 10 of each 100 chromosomes are selected for mutation operation and mutation-2, 1% means 1 of each 100 demands from a chromosome which is selected by mutation-1 will be chosen randomly for mutation operation. 

After crossover and mutation, the most important part of genetic algorithm is the fitness function which will after each iteration discard few of the solutions and promote the rest of the solutions for the next round. In our case we have three kind of fitness function based on our objective functions. Based on this fitness function score, different solutions are chosen for different cases. 

After running GA, the next part is to convert the chromosome into a data plane and then control a plane so that controller can make appropriate changes in the topology. Even though each chromosome indicates that if a link is used for a demand or not, the direction of the flow of the traffic is not defined. Therefore, the algorithm needs to take care the information regarding direction, when converting from chromosome to data plane and then control plane. The algorithm to retrieve the data plane Π and the control plane Γ is given in Algorithms 1 and 2 respectively. It simply corresponds to the following equation:

(9) $$\Pi_{k,v}=\chi_{k\times \left(v,w\right)}\times w$$

Algorithm 2 finds the minimal allocation satisfying the throughput demand gathered by the data plane. To note that the green policy, consists of shutdown **v** switches when $\delta_{\prod }^{v}=0$ and output ports **w** of switches **v** when $\delta_{\prod }^{v.w}=0$ and decreasing capacities of output ports **w** of switches **v** when $\Gamma_{v,w}<C_{v,w}.$ Finally, Algorithm 3 shows the overall algorithm for implementing GA. Here **eval()** function is evaluating the fitness of the chromosome.

**Algorithm 1.** Retrieval of data and control plane from chromosome.

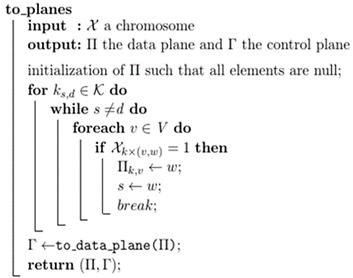



**Algorithm 2.** Computation of the minimum control plane from given data plane.

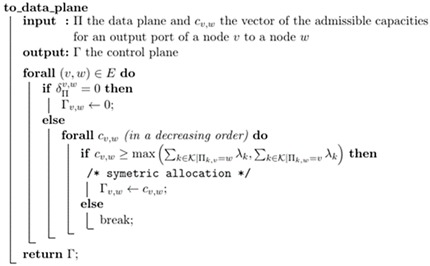



**Algorithm 3.** The Full Algorithm.

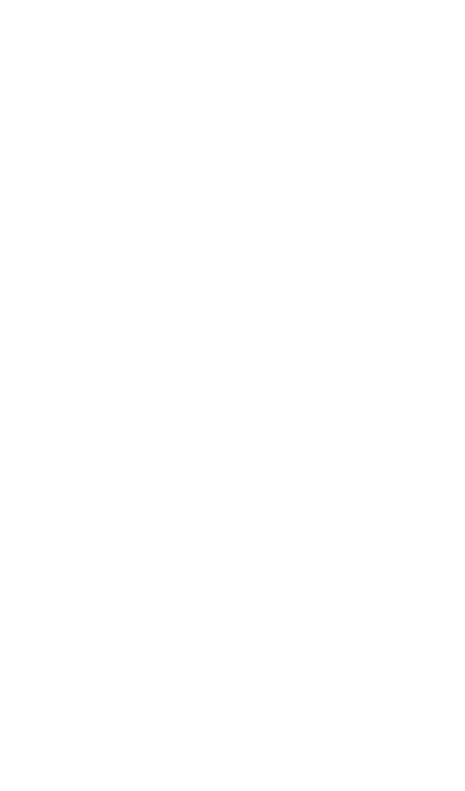



## 5. Evaluation of the System

### 5.1. Experiment Setup

For evaluating the performance of the system, the topology of the GÉANT network infrastructure has been considered. The GÉANT project operates the European network for the research and education community. GÉANT has 22 nodes and each node of GÉANT represents a different country. Because of that, the values of CEF and NRE will be different for each node. The network structure and the traffic has been taken from SNDlib [40] published in 2005. However, in order to keep the data compatible with the network equipment as it is now using 100-gbps optical fiber, network’s overall traffic has also increased significantly. From [41,42] it can be concluded that, during the last decade or so, traffic has increased almost 28 times. In order to keep up with the times, the real data is been multiplied by the increased traffic coefficient. Experiments are conducted with a set of 25 randomly generated demands. The demand sizes are randomly distributed. Network detail summary can be seen in Table 1. 

For power measurement, values are taken from [5,43]. For the links, power consumption of the optical transmission networking (OTN) layer interfaces per port has been considered. Power measurement values are given in Table 2. The values of carbon emission factor and non-renewable energy usage percentage have been taken from [44,45,46]. The details of all the national characteristics in terms of CEF and NRE are shown in Appendix B Table A2.

Lastly, the genetic algorithm has five factors, namely run time, population size, crossover rate, and two types of mutation rate. In order to run the experiment, we have tuned these values. In our work, we have fine-tuned these parameters using design of experiments. For this paper we are using the following values for the parameters of genetic algorithm in all the experiments: run time 30 s, population size 40, crossover rate 40%, mutation-1 rate 80% and mutation-2 rate 4%. The process of selecting the parameter values is briefly described in Appendix A.

### 5.2. Result Analysis

**Figure 7:**
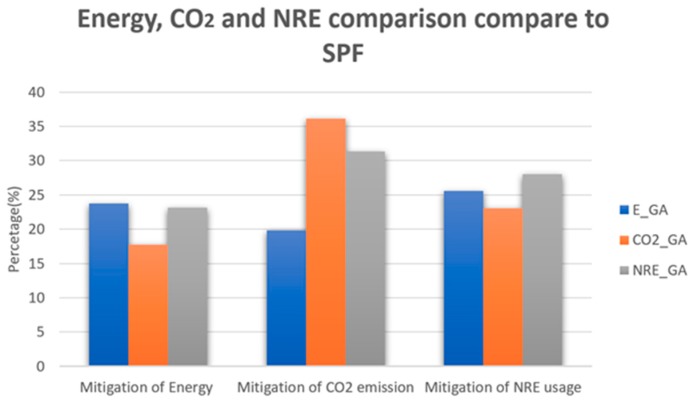
Comparison between three algorithms.

A set of experiments has been conducted to analyze the performance of the three different objective functions. The results are compared with shortest path first (SPF) with the same green policies that our algorithm has been considered. Twenty five demands are randomly taken for the experiment as we want to see how the algorithm uses the two green policies according to the objective function. This is because, if the number of demands are higher, then all the nodes of the topology will be involved as either source or destination, then all the nodes must be kept turned on and there will be no scope to analyze the performance of the algorithm in terms of first green policy where nodes will be shut down when not in use. Figure 7 shows the graph which gives the comparison of three different approaches. As we can see, every algorithm outperforms other two when their minimizing factor has been chosen. 

When energy is considered as the minimizing factor, that means for our EAR with GA (E_GA), we have maximum energy savings compared to the other two approaches. EAR saves around 24% of energy consumption compared to SPF for this scenario, whereas both approaches of PAR (CO_GA and NRE_GA) save around 18% and 23% energy consumption, respectively. However, when comes to reducing the CO_2_ emissions, both CO_2__GA and NRE_GA reduce an appreciable amount compared to E_GA. CO_2__GA reduces 36% of the CO_2_ emissions compared to SPF, whereas, NRE_GA and E_GA are able to reduce them by 31% and 20%, respectively. Even though our EAR (E_GA) has outperformed SPF by a large margin in all three aspects, in terms of reducing CO_2_ emissions both pollution-based algorithms performs better. In fact, when the optimizing function focuses on reducing CO_2_ emissions CO_2__GA reduces CO_2_ emissions by almost double compared to E_GA. Lastly, while for reducing non-renewable energy usage NRE_GA performs better than the other two. NRE_GA reduces the non-renewable energy usage by 28% compared to the shortest path. Table A3 in Appendix B shows the all obtained results for SPF and the other three algorithms. In contains country-wise consumption energy values, CO_2_ and non-renewable energy for each algorithm in order to understand the results more clearly. All these results raise the question, as the goal of green networking is to reduce the carbon footprint from the environment, then how much effective will designing a green network by only considering energy efficiency be. Even if a solution focusing on reducing CO_2_ emissions consumes more energy but if the total CO_2_ emissions of the system are lower that means the system is consuming more green energy than brown energy (energy sources producing a higher amount of CO_2_). 

Based only on the consumption values used in the paper, Table 3 provides an annual emission of energy CO_2_ and non-renewable energy. Here, at first, we have shown that if no green policy is used then what the total emissions will be and then the consumption values for shortest path first and our three algorithms are given. These values only reflect the emissions due to the devices and links. However, the actual amount will be much higher considering the building, cooling and many other criteria.

**Figure 8:**
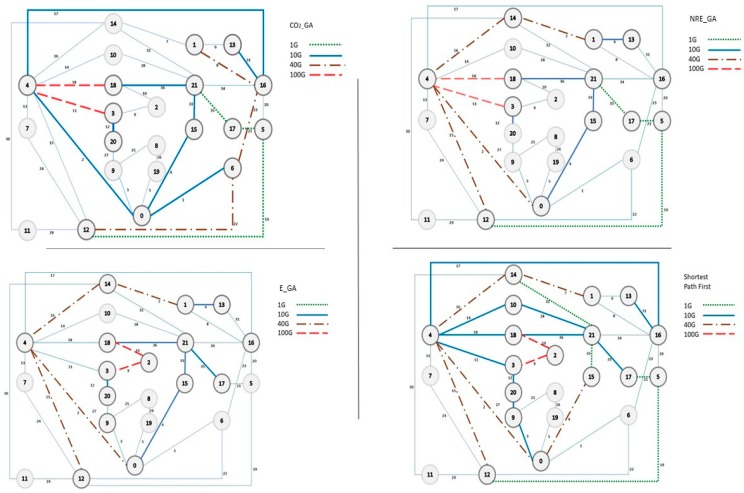
Topology for different algorithm.

Figure 8 gives the different topologies for different solutions. The first one in the top left gives the topology for SPF. As is clearly visible, the maximum number of nodes and links are used in this topology. Now if we analyze the differences between different topologies there are some interesting changes in the topology. The energy-based solution has not used node six and node sixteen which are Switzerland and France, and node 14 which is The Netherlands is used. For energy-based solution every node is considered as same as every node consumes same amount of energy, whereas, for the CO_2_-based solution the emissions could be completely different for the same number of nodes. As for our example, The Netherlands emits 130 times more CO_2_ gm per kw-h compared to Switzerland and six times more compared to France which has huge impact on the overall result. In the same way, node-2 (Poland) is used by E_GA whereas both of the PAR solutions avoid Poland as it has huge CEF and NRE usage percentage. These choices made by E_GA might reduce the overall energy consumption of the network, but at a cost of a high carbon emission.

Now, if we compare the solutions of the two PAR-based options, as mentioned above they both have avoided node-2 (Poland), however, for NRE_GA a majority of the traffic went through node-4 (Germany) and node-16 (France) is completely avoided, whereas for CO_2__GA a substantial amount of traffic went through France, even though it has very low carbon footprint. It is because NRE_GA only focuses on non-renewable energy percentage and as France uses nuclear power plants for producing electricity, it has very high non-renewable energy usage percentage. For example, for the same topology, let’s consider a demand from node 5 to node 14. There are several possible solutions. However, we will consider only the solutions which are shortest that means lowest number of hops. Now, there are four different possible paths. Table 4 gives a summary of all four shortest paths with carbon emission factors and non-renewable energy usage percentages. Now, here only the NRE and CEF values of intermediate nodes are considered. Based on the NRE_GA the optimal solution would be path-3 (with Italy and Germany) even though this path has one of the highest carbon emission factors, whereas, the optimal solution based on CEF would be path-2 but as this path has a maximum amount of NRE percentage therefore this will not be chosen by NRE_GA. 

Based on this analysis of the three algorithms, it can be said that even though the outcome of the algorithm depends on the demand set, it is very much a possibility that in order to achieve a sustainable solution focusing only on energy efficiency might not be a wise decision. Our result shows that for this particular scenario both of our algorithms outperform the energy-based solution in terms of reducing carbon emissions. Specially CO_2__GA reduces carbon emission by more than 20% compared to energy-based solution which is definitely a non-negligible amount.

### 5.3. Sustainability Discussion 

The three pillars of sustainability are a great means of explaining the complete sustainability problem. We have included all the three pillars into our system in order to provide a balanced sustainable solution. Our system provides the information about the throughput of different parts of the network. Whichever objective function is selected it gives an insight about the three parameters for measuring the status of the green networking, namely energy consumption, CO_2_ emissions and percentage of non-renewable energy usage, but as the goal of green networking is to reduce the carbon emissions, therefore the objective function minimizing the CO_2_ has utmost importance. As we can see, CO_2__GA reduces CO_2_ emissions by around 8% compared to our E_GA and by 40% compared to SPF with two green policies. These are definitely non-negligible values, however, a more tangible example might give a better understanding of the situation. For our scenario, if we compare the result of SPF and CO_2__GA from Table 3, in one year it can save up to 248 tons of CO_2_. This is in fact equivalent to the carbon emissions if a person were to make a round trip to JFK in New York from CDG in Paris by plane 381 times. Even when comparing with E_GA, the carbon savings is equivalent to 172 round trips by plane for the same source and destination. If we consider the savings in terms of money, even if we consider an average rate for cost of electricity production, our all three can save more than 25% of the cost compared to SPF. Lastly, the proposed system respects both the user traffic demand and the objective function choice and provides a data and control plane and hence integrates the social attribute of the sustainability.

## 6. Future Works

In our work we have proposed a solution which gives the required control plane and data plane for implementing it on an SDN platform. However, changes in the demand set result in a new topology and the new topology will be disseminated by the controller to the network. This dissemination process also requires energy, and hence has a carbon footprint. Therefore, for the future work we are planning to add a penalty system that will determine if a change in the network topology is necessary or not with a change in the demand set. In fact, this will control the frequency of network changes to an optimum level. Additionally, in this work all the nodes are equally treated whereas, that is not the case in reality. Our equation is flexible enough to add this variance into the system. Therefore, next step will be to adjust the equation accordingly and run the experiment for variable values for the node. Additionally, the carbon emissions and the non-renewable energy usage rate vary all the time. This is due to weather conditions, to the period of the day (night/day), to energy demand peaks requiring using for example more coal for producing electricity. Then, the idea is to include in the fitness function these variations in adding temporal factor in order to get more realistic results. In the same way, energy costs and its evolution could be integrated in the fitness function in order to cover the economy pillar of sustainable development. Finally, these variations (energy costs, carbon emitted for producing energy, etc.) could be estimated by predictive models for efficiently managing the changes of network topology in considering the penalty explained above. 

## 7. Conclusions

In this work, we have proposed a sustainable method for greening the Internet. We have introduced the term “pollution-aware routing” and it has been added to the classical energy-aware routing. While proposing this new term and introducing a new way of looking the problem of green networking we have at the same time tried to answer whether the question concerning only energy efficiency while trying to achieve green networking is enough or not. We have two different approaches, one works based on the carbon emission factor of the nodes whereas the second one works based on the non-renewable energy usage percentage. We show that our pollution-aware routing can have significant impact on CO_2_ emissions compared to energy-aware solutions. Our system provides a holistic approach towards attaining sustainability. At the same time, it provides a control plane and data plane so that the system can be implemented in a centralized system using SDN.

## Figures and Tables

**Table 1:** Network topology parameters.

Network	GÉANT
**Nodes**	22
**Links**	36
**Link type**	Full-Duplex
**Demand Structure**	(Source, Destination, Throughput)
**Demand Type**	Aggregated (one demand request for one source destination couple)
**Bandwidth Capacity**	1 Gbps, 10 Gbps, 40 Gbps, 100 Gbps

**Table 2:** Power consumption values for node and links.

Type	Power in Watts
Static Node	10000
1-Gbps port	7
10-Gbps port	34
40-Gbps port	160
100-gbps port	360

**Table 3:** Energy, cost and CO_2_ emission analysis for a year.

Method	Energy (MWh)	CO_2_ (Tons)	Non-Renewable Energy (MWh)
Without any green policy	2154	1088	1470
SPF	1522	684	1074
E_GA	1160	548	799
CO_2__GA	1252	436	824
NRE_GA	1170	470	777

**Table 4:** Demand paths from node-5 to node-14.

No.	Paths	NRE% Sum of the Intermediate Nodes	CEF Sum of the Intermediate Nodes
1	5-16-4-14	1.535	0.742
2	5-16-1-14	1.659	0.294
3	5-12-4-14	1.337	1.082
4	5-12-11-14	1.602	1.15

1-Belgium, 4-Germany, 5-Spain, 11-Israel, 12-Italy, 14-Netherlands, 16-France.

## Appendix A

As our heuristic algorithm is based on genetic algorithm, we have to do an extensive set of experiments for tuning the parameters. Five parameters are needed to be adjusted which are time, population size, crossover percentage, mutation-1 percentage and lastly mutation-2 percentage. We have selected few fixed values for each parameter for design of experiment. Each value of each parameter is preliminarily tested before being used in DoE. In Table A1, different level of values for each parameter is given. All the parameters are tuned in order to maximize objective function.

**Table A1:** DoE parameter summary.

Factor	Values
**Time**	10 s, 20 s, 30 s
**Population Size**	20, 40, 80
**Crossover Percentage**	20%, 40%, 80%
**Mutation-1 Percentage**	20%, 40%, 80%
**Mutation-2 Percentage**	4%, 8%, 12%

Running the full factorial experiment on our parameters using the above mentioned settings, provides us significant information about the parameter tuning. For both 25 demands and 400 demands DoE shows similar kinds of patterns. From these experiments we have achieved an optimum setting for all the genetic algorithm parameters which are, run time 30s, population size 40, crossover rate 40%, mutation-1 rate 80% and mutation-2 rate 4%.

## Appendix B

Table A2 shows the national characteristics in terms of carbon emission factor and non-renewable energy usage factor.

**Table A2:** List of nodes of the GEANT network with carbon and non-renewable energy factors.

Node No.	Country	CEF	NRE Factor
0	Austria	0.176	0.257
1	Belgium	0.224	0.834
2	Poland	1.196	0.863
3	Czech Republic	0.938	0.85
4	German	0.672	0.71
5	Spain	0.342	0.619
6	Switzerland	0.003	0.602
7	Greece	1.921	0.726
8	Croatia	0.386	0.348
9	Hungary	0.589	0.899
10	Ireland	0.521	0.753
11	Israel	0.74	0.975
12	Italy	0.41	0.327
13	Luxembourg	0.276	0.792
14	Netherlands	0.413	0.879
15	United States	0.547	0.72
16	France	0.07	0.825
17	Portugal	0.4	0.465
18	Sweden	0.023	0.429
19	Slovenia	0.578	0.694
20	Slovakia	0.282	0.755
21	United Kingdom	0.508	0.721

Table A3 shows the obtained results for each countries’ consumption of energy CO_2_ and non-renewable energy for our scenario.

**Table A3:** Countrywise energy, CO_2_ and non-renewable energy consumption values.

	Algorithm	SPF			GA_E			GA_CO_2_			GA_NRE		
Country		Energy (Watt)	CO_2_ (gCO_2_/kWh)	NRE (Watt)	Energy (Watt)	CO_2_ (gCO_2_/kWh)	NRE (Watt)	Energy (Watt)	CO_2_ (gCO_2_/kWh)	NRE (Watt)	Energy (Watt)	CO_2_ (gCO_2_/kWh)	NRE (Watt)
Austria		10354	1822.30	2660.97	10194	1794.14	2619.85	10102	1777.95	2596.21	10194	1794.14	2619.85
Belgium		10160	2275.84	8473.44	10194	2283.45	8501.79	10160	2275.84	8473.44	10194	2283.45	8501.79
Poland		10720	12821.1	9251.36	10720	12821.1	9251.36	0	0	0	0	0	0
Czech Republic		10428	9781.46	8863.8	10294	9655.77	8749.9	10394	9749.57	8834.9	10394	9749.57	8834.9
German		10616	7133.95	7537.36	10480	7042.56	7440.8	10788	7249.53	7659.48	11200	7526.4	7952
Spain		10014	3424.78	6198.66	0	0	0	10014	3424.78	6198.66	10014	3424.78	6198.66
Switzerland		0	0	0	0	0	0	10354	31.062	4162.30	0	0	0
Greece		0	0	0	0	0	0	0	0	0	0	0	0
Croatia		0	0	0	0	0	0	0	0	0	0	0	0
Hungary		10068	5930.05	9051.13	0	0	0	0	0	0	0	0	0
Ireland		10068	5245.42	7581.20	0	0	0	0	0	0	0	0	0
Israel		0	0	0	0	0	0	0	0	0	0	0	0
Italy		10167	4168.47	6374.70	10160	4165.6	6370.32	10167	4168.47	6374.70	10167	4168.47	6374.70
Luxembourg		10034	2769.38	7946.92	10034	2769.38	7946.92	10034	2769.38	7946.92	10034	2769.38	7946.92
Netherlands		10327	4265.05	9077.43	10320	4262.16	9071.28	0	0	0	10320	4262.16	9071.28
United States		10167	5561.34	8672.45	10068	5507.19	8588.00	10068	5507.19	8588.00	10068	5507.19	8588.00
France		10068	704.76	8306.1	0	0	0	10388	727.16	8570.1	0	0	0
Portugal		10041	4016.4	4669.06	10034	4013.6	4665.81	10014	4005.6	4656.51	10014	4005.6	4656.51
Sweden		10428	239.844	4473.61	10394	239.062	4459.02	10394	239.062	4459.02	10394	239.062	4459.02
Slovenia		0	0	0	0	0	0	0	0	0	0	0	0
Slovakia		10068	2839.17	7601.34	10034	2829.58	7575.67	10034	2829.5	7575.67	10034	2829.5	7575.67
United Kingdom		10116	5138.9	7293.63	10102	5131.816	7283.542	10075	5118.1	7264.075	10075	5118.1	7264.075
